# U-Shaped Association Between β-Carotene Intake and Suicidal Ideation in Cancer Survivors: Evidence from a Nationally Representative Sample

**DOI:** 10.3390/nu18101567

**Published:** 2026-05-14

**Authors:** Hyejin Tae, Tae-Suk Kim

**Affiliations:** 1 Stress Clinic, Health Promotion Center, Seoul St. Mary’s Hospital, College of Medicine, The Catholic University of Korea, Seoul 06591, Republic of Korea; hyejin.tae@catholic.ac.kr; 2 Department of Psychiatry, Seoul St. Mary’s Hospital, The Catholic University of Korea, Seoul 06591, Republic of Korea

**Keywords:** β-carotene, suicidal ideation, mental health, nutrition, cancer survivors, micronutrients, dose–response, non-linear association

## Abstract

Background/Objectives: Nutritional factors, including β-carotene with antioxidant and anti-inflammatory properties, are increasingly recognized for their role in mental health. However, population-based evidence on micronutrient intake and suicidal ideation among cancer survivors remains limited. This study investigated the association between dietary β-carotene intake and suicidal ideation among cancer survivors using nationally representative data. Methods: We conducted a cross-sectional analysis of 698 adult cancer survivors from the 2014–2020 Korea National Health and Nutrition Examination Survey. Suicidal ideation was assessed using the ninth item of the Patient Health Questionnaire. Dietary β-carotene intake was estimated using a 24-h dietary recall. Multivariable logistic regression models were applied with adjustment for sociodemographic, lifestyle, clinical, and dietary factors. Restricted cubic spline models were used to examine non-linear dose–response relationships. Subgroup analyses were performed by age, sex, and time since diagnosis. Results: Among participants, 6.3% reported suicidal ideation. Higher β-carotene intake was associated with lower odds of suicidal ideation (adjusted OR 0.68 per 1000 µg increase, 95% CI 0.50–0.93). Restricted cubic spline analyses revealed a significant U-shaped association (*p* for non-linearity = 0.030), indicating that the risk of suicidal ideation was lowest at an intermediate intake level (approximately 6000 µg/day). Stronger associations were observed among younger individuals and those ≥5 years post-diagnosis, with no significant interaction by sex. Conclusions: Dietary β-carotene intake showed a non-linear association with suicidal ideation, suggesting that both insufficient and excessive intake are associated with higher odds of suicidal ideation. These findings suggest the potential importance of optimal micronutrient balance in mental health and suggest that dietary factors may be associated with suicidal ideation among cancer survivors.

## 1. Introduction

Suicide remains a critical public health concern worldwide. According to the World Health Organization (WHO), it claims more than 720,000 lives annually and imposes a substantial socioeconomic burden [1]. In the United States alone, the annual economic cost of suicide has been estimated at approximately 484 billion dollars [2]. Cancer survivors represent a particularly vulnerable group in terms of mental health. The complex interplay of physical, emotional, and psychosocial stressors following a cancer diagnosis significantly increases the risk of psychiatric disorders such as depression, anxiety, and insomnia [3]. As a result, cancer survivors exhibit a higher prevalence of suicidal ideation compared to the general population [4]. Suicidal thoughts in this population can negatively impact treatment adherence, persistence, and overall prognosis [5].

In South Korea, suicide remains a leading cause of death among individuals aged 10 to 39 years, with an age-standardized suicide rate of 27.3 per 100,000 population in 2023—more than twice the OECD average [6]. Moreover, Korean cancer survivors have been shown to experience a 1.5–2 times higher suicide risk than the general population [7], underscoring the need for targeted preventive strategies in this group. Identifying modifiable factors that may reduce suicidal ideation in cancer survivors is therefore an important public health priority.

There has been growing interest in the relationship between dietary factors and mental health. Among various micronutrients, β-carotene—a provitamin A carotenoid well-characterized for its antioxidant and anti-inflammatory properties—has emerged as a biologically plausible candidate linking nutrition and mental health. β-carotene reduces oxidative stress by neutralizing reactive oxygen species and downregulates pro-inflammatory cytokines such as interleukin-6 (IL-6) and tumor necrosis factor-alpha (TNF-α), which are implicated in mood disorders and neuroinflammation [8,9]. In addition, it promotes neuroplasticity by enhancing the expression of brain-derived neurotrophic factor (BDNF), a key molecule in the regulation of emotion and cognition [10]. Given that oxidative stress, inflammation, and impaired neuroplasticity are implicated in both depression and suicidality, β-carotene may be particularly relevant to mental health outcomes.

Empirical studies have reported inverse associations between β-carotene intake and the prevalence of depression [11,12]. Beyond mood disorders, higher β-carotene levels have also been linked to a reduced risk of suicidal ideation. In a cross-sectional study of 7335 U.S. adults, higher serum β-carotene concentrations were associated with a lower risk of suicidal ideation [13]. Similarly, an analysis of Korean survey data found that higher dietary carotene intake was associated with a decreased risk of suicidal ideation among females [14]. However, evidence in cancer survivors remains limited, despite their distinct psychological vulnerability and potential alterations in nutritional status following diagnosis and treatment.

Therefore, this study aimed to investigate the association between dietary β-carotene intake and suicidal ideation in a nationally representative sample of Korean cancer survivors. Our group previously reported an inverse association between dietary riboflavin intake and suicidal ideation using KNHANES data [15], and the present study extends this line of research by focusing on β-carotene in cancer survivors.

## 2. Materials and Methods

### 2.1. Data Source and Study Population

This study utilized data from the Korea National Health and Nutrition Examination Survey (KNHANES), a nationwide, population-based survey conducted by the Korea Disease Control and Prevention Agency (KDCA) to assess the health and nutritional status of the Korean population. KNHANES is composed of three primary components: a health interview survey, a health examination survey, and a nutrition survey. The health interview survey collects information on socioeconomic status, health-related behaviors, quality of life, and healthcare utilization. The health examination survey includes anthropometric measurements and biochemical and clinical profiles related to non-communicable diseases. The nutrition survey assesses dietary intake and eating behaviors. These components are conducted by trained staff members, including physicians, medical technicians, and dietitians, using standardized protocols to ensure data reliability and validity [15]. The KNHANES employs standardized survey protocols and questionnaires across survey cycles, ensuring comparability of data over time; detailed information is available in the KNHANES documentation [16].

**Figure 1:**
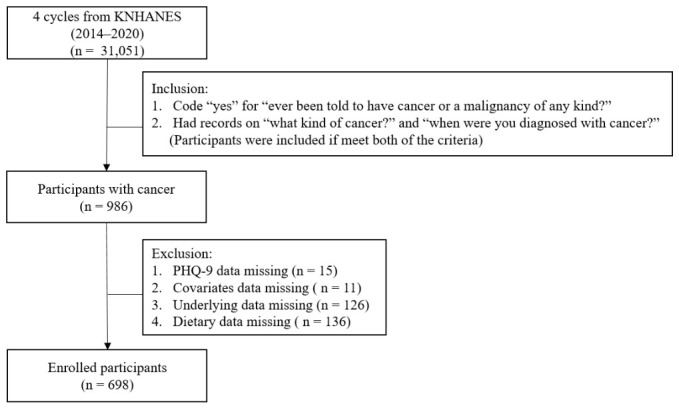
Flowchart of participant selection and cancer type distribution among cancer survivors in the Korea National Health and Nutrition Examination Survey (KNHANES), 2014–2020.

For this analysis, data from the sixth to eighth cycles of KNHANES (2014–2020) were used, encompassing a total sample size of 31,051 individuals. From an initial sample of 31,051 individuals, 986 participants with a physician-diagnosed cancer were identified. After excluding those with missing data on PHQ-9, covariates, clinical variables, or dietary intake, 698 participants were included in the final analysis. The detailed selection process is presented in Figure 1. All KNHANES protocols were approved by the Institutional Review Board (IRB) of the Korea Disease Control and Prevention Agency. Participants provided written informed consent prior to their participation in the survey. The specific IRB approval numbers for the survey cycles included in this study are as follows: 2013-12EXP-03-5C (19 May 2014), 2018-01-03-P-A (5 January 2018), and 2018-01-03-2C-A (10 May 2019).

In this study, cancer survivorship was defined in accordance with widely accepted epidemiological standards—namely, individuals who reported a physician diagnosis of cancer and were alive at the time of survey participation. This approach aligns with the definition first proposed by Mullan [17], and later adopted by the National Cancer Institute and other expert bodies, which considers an individual a cancer survivor from the time of diagnosis through the remainder of life.

### 2.2. Assessment of β-Carotene Intake

Dietary intake data, including β-carotene consumption, were collected through a single 24-h dietary recall interview conducted by trained dietitians as part of KNHANES. Participants were asked to recall all foods and beverages consumed in the previous 24 h, including portion sizes, preparation methods, and, when applicable, brand names. This method has been widely used in large-scale nutritional epidemiology and validated for assessing nutrient intake in the Korean population [18].

The collected dietary data were analyzed using the Korean Food Composition Table (KFCT), developed and regularly updated by the Rural Development Administration. The KFCT provides detailed information on the nutrient content of foods commonly consumed in Korea, including β-carotene levels [19]. Daily β-carotene intake for each participant was estimated by applying the KFCT to the 24-h recall data.

### 2.3. Assessment of Psychological Symptoms

Depressive symptoms in this study were evaluated using the Patient Health Questionnaire-9 (PHQ-9), a widely used and validated screening instrument for depressive symptoms in both clinical and community populations. The PHQ-9 consists of nine items assessing the frequency of depressive symptoms over the past two weeks, with responses rated on a four-point Likert scale ranging from 0 (“not at all”) to 3 (“nearly every day”), yielding a total score between 0 and 27. In accordance with previous studies using Korean samples, a cutoff score of 10 or higher was used to define clinically relevant depressive symptoms [20].

Suicidal ideation was assessed using item 9 of the PHQ-9, which specifically asks about the presence of thoughts related to death or self-harm during the preceding two weeks. Participants who responded with any response other than “not at all”—including “several days,” “more than half the days,” or “nearly every day”—were classified as having experienced suicidal ideation [21].

### 2.4. Assessment of Cancer History and Classification

Cancer-related information was obtained through self-reported responses in the KNHANES health interview survey. Participants were asked whether they had ever been diagnosed with cancer by a physician. For those who responded affirmatively, additional information was collected regarding the type of cancer, age at diagnosis, and current cancer status. Cancer types were categorized into predefined groups (stomach, liver, colorectal, breast, cervical, lung, thyroid, and other cancers) based on standardized KNHANES classifications [16,22]. Time since cancer diagnosis was calculated using the reported age at diagnosis and current age and was included as a covariate in the analysis. Although cancer history was self-reported, previous studies have demonstrated acceptable agreement between self-reported diagnoses and medical records for major chronic diseases, including cancer [23].

### 2.5. Assessment of Covariates

In this study, a range of sociodemographic and behavioral characteristics were included as covariates. Sociodemographic variables comprised age, sex, marital status, educational attainment, and household income. Education level was categorized into four groups: less than elementary school, middle school graduate, high school graduate, and college degree or higher. Household income was divided into quartiles: low, lower-middle, upper-middle, and high.

Behavioral factors included smoking status, alcohol consumption, and physical activity. Current smokers were defined as individuals who had smoked at least 100 cigarettes in their lifetime and continued to smoke at the time of the survey. Alcohol users were identified as those who reported drinking alcohol at least once per month over the past year. Physical activity was assessed separately for aerobic and resistance exercise. Participants were considered physically active if they reported engaging in aerobic activity at least five times per week for a minimum of 30 min per session. Otherwise, they were classified as physically inactive. Resistance exercise was measured using a question about participation in muscle-strengthening activities such as push-ups, sit-ups, or weightlifting.

Anthropometric and clinical measurements were also included. Body mass index (BMI) was calculated by dividing weight in kilograms by height in meters squared (kg/m^2^). Obesity was defined as a BMI ≥ 25 kg/m^2^ according to criteria recommended for Asian populations [24]. Hypertension was defined by systolic blood pressure ≥ 140 mmHg, diastolic blood pressure ≥ 90 mmHg, current use of antihypertensive medications, or self-reported diagnosis [25]. Diabetes was determined based on fasting plasma glucose ≥ 126 mg/dL, use of antidiabetic medications, or physician diagnosis [26]. Hyperlipidemia was identified if participants met any of the following criteria: total cholesterol ≥ 240 mg/dL, triglycerides ≥ 150 mg/dL, HDL cholesterol ≤ 40 mg/dL, LDL cholesterol ≥ 130 mg/dL, or current use of lipid-lowering medications. Blood samples were obtained after a minimum of 8 h of fasting. Fasting glucose and lipid profiles were measured using enzymatic colorimetric methods with the Labospect 008AS autoanalyzer (Hitachi, Tokyo, Japan).

### 2.6. Statistical Analyses

All statistical analyses accounted for the complex sampling design of KNHANES, which incorporates stratification, clustering, and sample weights to ensure national representativeness and unbiased variance estimation. Descriptive characteristics of participants were summarized by quartiles of β-carotene intake. Continuous variables were expressed as weighted means with standard deviations (SD), and categorical variables as weighted frequencies and percentages. Group differences were assessed using survey-weighted one-way analysis of variance (ANOVA) for continuous variables and the chi-square test for categorical variables.

To examine the association between β-carotene intake and suicidal ideation, multivariable logistic regression models were constructed. β-carotene intake was modeled as a continuous variable per 1000 μg/day increase to facilitate interpretation of effect estimates. In addition, β-carotene intake was categorized into quartiles for trend analysis, and the *p* for trend was calculated by modeling the median value of each quartile as a continuous variable. Model 1 was unadjusted; Model 2 adjusted for age and sex; and Model 3 further included marital status, education, household income, smoking status, alcohol use, aerobic and resistance exercise, BMI, depression, hypertension, diabetes, dyslipidemia, energy intake, and micronutrients (vitamins A, B1, B2, B3, C, D, E, and folate), as well as time since cancer diagnosis. To minimize residual confounding, we additionally adjusted for micronutrients that share antioxidant and neuroprotective pathways with β-carotene and have been independently linked to depression and suicidality [12]. Potential multicollinearity was assessed using variance inflation factors (VIF). To evaluate potential non-linear dose–response relationships, restricted cubic spline (RCS) regression models were applied. RCS models were fitted using three knots at fixed percentiles of β-carotene intake. Subgroup and stratified analyses were conducted by sex, age, and cancer duration, with interaction terms used to assess effect modification. All analyses were performed using R statistical software (version 4.5.0; R Foundation for Statistical Computing, Vienna, Austria), with statistical significance defined as a two-sided *p*-value < 0.05.

## 3. Results

### 3.1. Baseline Characteristics of the Study Population

Among the 698 cancer survivors included in the study, 44 participants (6.3%) reported experiencing suicidal ideation. Baseline characteristics of the study population according to suicidal ideation status are presented in Table 1. Participants with suicidal ideation were significantly older than those without suicidal ideation (67.6 vs. 61.8 years, *p* < 0.001) and more likely to have lower educational attainment and household income (*p* < 0.001 for both). In terms of health behaviors, current smoking (*p* = 0.033) and lower levels of aerobic physical activity (*p* = 0.001) were more common among individuals with suicidal ideation. Clinically, the prevalence of hypertension (*p* = 0.019) and depression (*p* < 0.001) was significantly higher, and hyperlipidemia showed a borderline difference between groups (*p* = 0.056). Nutritionally, individuals with suicidal ideation had significantly lower intake of energy, protein, fat, vitamin D, vitamin E, riboflavin, niacin, folate, retinol, and vitamin C (all *p* < 0.05). While β-carotene intake was also lower in the suicidal ideation group, the difference did not reach statistical significance (*p* = 0.089).

### 3.2. Association Between β-Carotene Intake and Suicidal Ideation

Multivariable logistic regression analyses were conducted to evaluate the association between β-carotene intake and suicidal ideation (Table 2). In the fully adjusted model (Model 3), higher β-carotene intake was significantly associated with lower odds of suicidal ideation in the continuous model (OR = 0.68; 95% CI: 0.50–0.93; *p* = 0.015); however, this relationship did not follow a clear linear dose–response pattern, suggesting a potential non-linear association. This inverse relationship was generally consistent across Models 1 and 2. Multicollinearity diagnostics revealed no evidence of severe collinearity among covariates (all VIFs < 10; Supplementary Table S1), confirming the stability of the estimated associations even after adjusting for multiple dietary factors.

Depression was the strongest risk factor (OR = 20.04, 95% CI: 8.99–44.67, *p* < 0.001), followed by current smoking (OR = 2.60, 95% CI: 1.24–5.45, *p* < 0.05). Notably, the inverse association of β-carotene intake was comparable in magnitude to that of higher educational attainment (OR = 0.67, 95% CI: 0.47–0.95, *p* < 0.05), suggesting a potential association with dietary factors in mental health among cancer survivors. The full multivariable regression results are visualized in Supplementary Figure S1. The overall model was statistically significant (model χ^2^ test, *p* < 0.001), indicating adequate model fit.

Multivariable logistic regression analyses stratified by age revealed a statistically significant inverse association between β-carotene intake and suicidal ideation among participants younger than 60 years (OR = 0.58, 95% CI: 0.38–0.89), whereas no significant association was found in those aged 60 years or older (OR = 0.79, 95% CI: 0.57–1.11). When stratified by time since cancer diagnosis, β-carotene intake remained significantly associated with a reduced risk of suicidal ideation in individuals diagnosed five or more years earlier (OR = 0.68, 95% CI: 0.50–0.93), while no significant association was observed in those diagnosed within the past five years (OR = 1.16, 95% CI: 0.79–1.70). In contrast, stratified analyses by sex revealed no statistically significant associations in either men or women. No statistically significant interactions were observed across these subgroup analyses (all *p* for interaction > 0.05).

To further explore this relationship, a linear trend analysis was performed using quartiles of β-carotene intake, with the lowest quartile (Q1) as the reference. The odds ratios (95% CI) for Q2, Q3, and Q4 were 0.64 (0.30–1.37), 0.53 (0.23–1.24), and 0.84 (0.38–1.86), respectively. The *p*-value for trend was 0.429, indicating no evidence of a linear trend across quartiles and suggesting a possible non-linear relationship, with some variability in estimates depending on model parameterization.

### 3.3. Analysis of Restricted Cubic Spline (RCS) Regression

**Figure 2:**
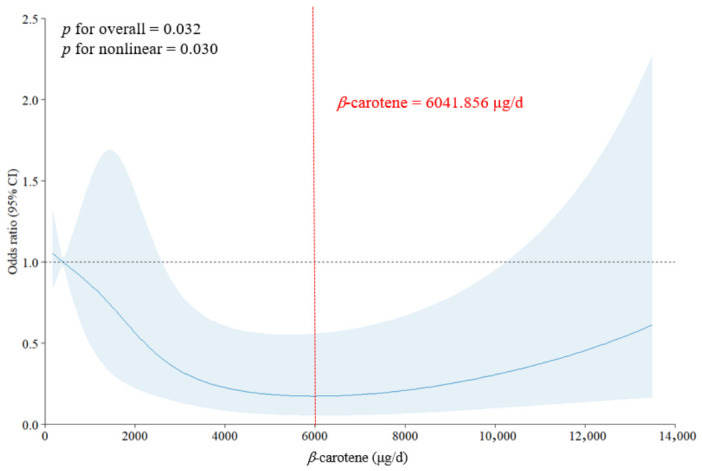
Nonlinear association between dietary β-carotene intake and suicidal ideation among cancer survivors. Results from restricted cubic spline regression analysis. The solid line represents adjusted odds ratios, and the shaded area indicates 95% confidence intervals. The horizontal dashed line indicates an odds ratio of 1.0, and the vertical dashed line indicates the β-carotene intake level associated with the lowest estimated odds ratio (6041.856 μg/d). The widening of the confidence intervals at the high-intake end likely reflects smaller sample sizes in this range, as illustrated in the rug plot (Figure S3).

To examine the potential non-linear dose–response relationship between β-carotene intake and suicidal ideation, we employed restricted cubic spline (RCS) regression modeling. The analysis revealed a statistically significant non-linear association (*p* for non-linearity = 0.030), characterized by a U-shaped curve (Figure 2). The lowest estimated risk of suicidal ideation was observed at moderate levels of β-carotene intake, approximately around 6000 μg/day.

To further investigate this pattern, subgroup-specific RCS analyses were conducted by sex, age group, and time since cancer diagnosis (Supplementary Figure S2). A similar U-shaped pattern was observed across subgroups, with significant non-linearity found for sex (*p* = 0.004), age categories (*p* = 0.008), and cancer duration groups (*p* = 0.008).

## 4. Discussion

In this nationally representative study of Korean cancer survivors, we identified a significant inverse association between dietary β-carotene intake and suicidal ideation. Participants reporting suicidal ideation were more likely to be older, socioeconomically disadvantaged, physically inactive, and clinically burdened with hypertension. Multivariable analyses confirmed that higher β-carotene intake was associated with reduced odds of suicidal ideation, even after adjustment for a wide range of sociodemographic, behavioral, clinical, and dietary factors. This association remained significant in individuals under the age of 60 and those with a longer duration since cancer diagnosis, but not in older adults or those more recently diagnosed. Furthermore, restricted cubic spline modeling revealed a significant U-shaped association, with the lowest estimated risk observed at moderate levels of β-carotene intake, broadly corresponding to approximately 6000 μg/day. These results were consistent across subgroups, suggesting consistency of the observed association. Taken together, these findings provide observational evidence of a non-linear relationship between dietary β-carotene intake and suicidal ideation in cancer survivors, extending prior findings from general populations and highlighting the potential importance of nutritional factors in survivorship mental health.

These results build on prior evidence linking antioxidant-rich diets to improved mental health outcomes in vulnerable populations and underscore the potential relevance of micronutrient-related strategies in the psycho-oncological care of cancer survivors. Several interrelated neurobiological mechanisms may help explain the observed inverse association between β-carotene intake and suicidal ideation in cancer survivors. Specifically, cancer survivors often exhibit heightened systemic inflammation and persistent oxidative stress even years after treatment, characterized by elevated pro-inflammatory markers that are closely linked to the pathophysiology of depression and suicidality [27]. β-carotene, a potent antioxidant, may help attenuate oxidative stress—a well-established contributor to affective dysregulation and suicidality—by scavenging reactive oxygen species and preserving redox balance within the central nervous system [28]. These effects are particularly critical in emotion-regulating regions such as the prefrontal cortex and hippocampus, which are highly susceptible to oxidative damage and structural alterations in individuals with suicidal behavior [29]. β-carotene has been suggested to suppress pro-inflammatory cytokines, including IL-6 and TNF-α, which are elevated in suicidality and exacerbate neuroinflammatory cascades via microglial activation and stimulation of the kynurenine pathway, leading to the accumulation of neurotoxic metabolites such as quinolinic acid [30,31]. In addition, β-carotene has been proposed to enhance brain-derived neurotrophic factor (BDNF) expression, a key regulator of neuronal survival and synaptic plasticity, whose deficits have been linked to increased suicidal ideation [32,33]. Taken together, these pathways suggest three primary mechanisms through which β-carotene may be associated with lower suicidality: (1) attenuation of oxidative stress, (2) suppression of neuroinflammatory cascades, and (3) enhancement of neurotrophic signaling via BDNF. These convergent processes suggest that β-carotene may be associated with biological pathways relevant to mood and suicidality, while underscoring the need for biomarker-based studies to validate these mechanisms in cancer populations.

Importantly, our restricted cubic spline (RCS) analysis revealed a U-shaped association, suggesting that the estimated risk of suicidal ideation was lowest within an intermediate range of β-carotene intake, approximately around 6000 μg/day, although this value should be interpreted as an approximate range rather than a precise optimal threshold. This level of intake may be achievable through the moderate consumption of β-carotene-rich foods, such as carrots, spinach, or sweet potatoes [34]. This non-linear pattern suggests that both lower and higher levels of β-carotene intake may be associated with a higher likelihood of suicidal ideation. However, estimates at the higher intake range should be interpreted with caution, as they were based on relatively limited data and showed wider confidence intervals. While β-carotene is widely recognized for its antioxidant properties, prior studies have suggested that high-dose supplementation may under certain conditions exert pro-oxidant effects, particularly in populations exposed to elevated oxidative stress such as smokers or individuals with chronic inflammation or mitochondrial dysfunction [35,36]. In these contexts, β-carotene may undergo oxidative cleavage, producing reactive aldehydes and carotenoid radical species that could amplify lipid peroxidation and cellular damage [35]. Furthermore, excess β-carotene has been hypothesized to disrupt retinoid homeostasis, which may in turn influence neuroendocrine signaling and mood regulation [37]. From a neuropsychiatric standpoint, such mechanisms have been proposed to affect glial function, neurotransmitter systems, and synaptic plasticity in mood-related brain regions [28,29]. Accordingly, our findings suggest that an optimal range, rather than uniformly higher intake, may be relevant; however, these findings should be interpreted as reflecting a non-linear association rather than a consistent linear trend.

Subgroup analyses further revealed a differential impact of β-carotene intake on suicidal ideation according to age and time since cancer diagnosis. The stronger inverse association observed among younger survivors and individuals more than five years post-diagnosis may reflect greater neuroplastic and metabolic responsiveness to nutritional factors, as well as increased psychological stabilization following the acute cancer experience. Younger adults typically exhibit higher baseline BDNF levels and more robust antioxidant defenses, which may potentiate the beneficial effects of micronutrients like β-carotene on brain function and emotional regulation [38]. In contrast, older adults often experience age-related declines in neurogenesis, immune function, and nutritional absorption, which can attenuate the neuropsychiatric impact of dietary interventions [39]. Moreover, long-term survivors are more likely to have transitioned into the re-integration or chronic survivorship phase, characterized by a shift from acute illness-related stressors to more manageable psychosocial adaptation, thereby creating a more receptive context for preventive mental health strategies [40]. On the other hand, among recently diagnosed patients, the psychological impact of a new cancer diagnosis—coupled with the burden of active treatment and comorbid physical symptoms—may eclipse the subtle effects of diet on mental health outcomes [41]. These findings suggest that dietary interventions, including micronutrient-based strategies, may yield greater mental health benefits when tailored to specific survivorship stages and biological profiles, particularly among younger or long-term survivors who possess a more favorable terrain for nutritional modulation.

Several limitations must be acknowledged. First, the cross-sectional design precludes causal inference, and reverse causality cannot be excluded, as individuals with suicidal ideation may modify their dietary behaviors. Second, dietary intake was assessed using a single 24 h recall, which may not adequately reflect habitual intake patterns and is subject to recall bias. Third, suicidal ideation was measured using a single PHQ-9 item, capturing its presence but not severity or clinical significance; thus, the findings reflect the occurrence of suicidal ideation rather than clinically actionable behavior. Fourth, serum β-carotene levels were unavailable, and intake from dietary supplements was not captured, potentially leading to exposure misclassification. Therefore, total β-carotene exposure may have been underestimated. Fifth, although the overall sample size was sufficient for primary analyses, the relatively small number of participants reporting suicidal ideation may have limited the statistical power and increased the risk of overfitting in fully adjusted and stratified models; accordingly, subgroup findings should be considered exploratory. Sixth, the sparse distribution of data at higher intake levels resulted in wider confidence intervals in the restricted cubic spline models. In addition, the direction of association varied across alternative model specifications, suggesting sensitivity to parameterization and potential instability due to limited events and correlated covariates; these findings should therefore be interpreted with caution. Seventh, residual confounding may remain due to unmeasured variables, including cancer-related factors (e.g., type, stage, treatment modality) and antidepressant use, as well as limitations in capturing overall diet quality. In addition, overall diet quality may not be fully captured by adjustment for total energy intake and selected micronutrients. Finally, although we adjusted for multiple dietary micronutrients to reduce confounding, several of these variables share biological pathways and dietary sources with β-carotene, raising the possibility of conceptual overadjustment. Accordingly, the observed associations may be conservative. Consistent with our previous KNHANES-based findings on riboflavin intake and suicidal ideation [15], the present results may support the role of broader antioxidant-related nutritional patterns in mental health. Nevertheless, β-carotene showed a distinct U-shaped nonlinear association in cancer survivors, suggesting potential biological relevance beyond overall dietary quality alone.

From a public health perspective, these results underscore the importance of incorporating nutritional assessments into comprehensive survivorship care plans. Despite growing recognition of the role of diet in mental health, nutritional screening remains underutilized in oncology and psychiatric settings [42]. Several studies have demonstrated that cancer survivors frequently experience micronutrient deficiencies, including carotenoids, which are rarely addressed in routine follow-up care despite their implications for mood and cognitive function [43]. β-carotene-rich foods—such as leafy greens, carrots, and sweet potatoes—are accessible and culturally acceptable dietary sources that may be associated with a lower likelihood of suicidal ideation among cancer survivors. Taken together, these findings suggest that nutritional factors may be associated with mental health outcomes among cancer survivors and may inform future research on dietary approaches in survivorship care.

## 5. Conclusions

In conclusion, our study suggests that dietary β-carotene intake is inversely associated with suicidal ideation among cancer survivors, with particularly stronger associations observed in younger individuals and those further along the survivorship trajectory. Notably, the association follows a U-shaped dose–response pattern, suggesting that an intermediate intake range may be associated with lower odds of suicidal ideation. These findings highlight dietary β-carotene as a potentially modifiable nutritional factor associated with mental health in cancer survivorship. Incorporating nutritional assessment and counseling into survivorship care programs may be useful for identifying individuals at higher likelihood of suicidal ideation. Further longitudinal and intervention studies are needed to confirm these findings, clarify temporal and causal relationships, and inform future research on dietary approaches in mental health among cancer survivors.

## Figures and Tables

**Table 1:** Baseline characteristics of cancer survivors according to suicidal ideation status.

Characteristic	No Suicidal Ideation, *n* (%)	With Suicidal Ideation, *n* (%)	*p*-Value
Participants	654	44	
Age, y	61.8 ± 12.5	67.6 ± 9.0	<0.001
Sex			0.530
Male	229 (35.0)	18 (40.9)	
Female	425 (65.0)	26 (59.1)	
Marital status			1.000
Married/Living with partner	636 (97.2)	43 (97.7)	
Widowed/Never married/Divorced/Separated	18 (2.8)	1 (2.3)	
Educational level			<0.001
Elementary or less	185 (28.3)	28 (63.6)	
Middle school	90 (13.8)	5 (11.4)	
High school	198 (30.3)	8 (18.2)	
College or more	181 (27.7)	3 (6.8)	
Household income			<0.001
Low	181 (27.7)	28 (63.6)	
Medium–low	152 (23.2)	7 (15.9)	
Medium–high	164 (25.1)	7 (15.9)	
High	157 (24.0)	7 (4.5)	
Smoking status			0.033
No	438 (67.0)	22 (50.0)	
Yes	216 (33.0)	22 (50.0)	
Drinking status			1.000
No	432 (66.1)	29 (65.9)	
Yes	222 (33.9)	15 (34.1)	
Aerobic exercise			0.001
<1 day/week	120 (18.3)	18 (40.9)	
1–3 days/week	172 (26.3)	9 (20.5)	
4–7 days/week	362 (55.4)	17 (38.6)	
Resistance exercise			0.625
<1 day/week	502 (76.8)	36 (81.8)	
1–3 days/week	75 (11.5)	3 (6.8)	
4–7 days/week	77 (11.8)	5 (11.4)	
Hypertension			0.019
Normal	225 (34.4)	12 (27.3)	
Prehypertension	163 (24.9)	5 (11.4)	
Hypertension	266 (40.7)	27 (61.4)	
Diabetes			0.610
Normal	330 (50.5)	19 (43.2)	
Prediabetes	192 (29.4)	14 (31.8)	
Diabetes	132 (20.2)	11 (25.0)	
Hyperlipidemia			0.056
No	481 (73.5)	26 (59.1)	
Yes	173 (26.5)	18 (40.9)	
Depression			<0.001
No	629 (96.2)	23 (52.3)	
Yes	25 (3.8)	21 (47.7)	
Time Since Cancer Diagnosis, years	8.3 ± 7.3	10.3 ± 9.9	0.194
Short (≤1 year)	69 (10.6)	3 (6.8)	0.681
Medium (>1–5 years)	217 (33.2)	14 (31.8)	
Long (>5 years)	368 (56.3)	27 (61.4)	
Body Mass Index, kg/m^2^	24.0 ± 3.2	23.9 ± 3.2	0.859
Daily Energy Intake, kcal/d	1751.5 ± 705.4	1464.6 ± 754.4	0.010
Carbohydrates, g/d	285.9 ± 111.6	254.8 ± 124.6	0.075
Protein, g/d	62.2 ± 30.2	45.3 ± 25.0	<0.001
Fat, g/d	34.6 ± 24.5	21.6 ± 16.7	<0.001
Vitamin D, μg/d	2.6 ± 3.9	1.5 ± 2.4	0.006
Vitamin E, mg α-TE/d	6.0 ± 3.5	4.5 ± 3.0	0.003
β-carotene, μg/d	3064.9 ± 3019.4	2259.5 ± 3269.7	0.089
Retinol, μg/d	110.4 ± 180.2	54.8 ± 79.2	<0.001
Thiamine, mg/d	1.2 ± 0.7	1.0 ± 0.7	0.036
Riboflavin, mg/d	1.4 ± 0.8	1.0 ± 0.7	<0.001
Niacin, mg/d	11.3 ± 6.1	8.2 ± 5.0	0.001
Folate, μg DFE	341.1 ± 174.6	280.3 ± 175.2	0.026
Ascorbic Acid, mg/d	66.3 ± 66.7	42.0 ± 38.1	<0.001

Note. Values are presented as mean ± standard deviation (SD) for continuous variables and number (percentage) for categorical variables. *p*-values were calculated using *t*-tests for continuous variables and chi-square or Fisher’s exact tests for categorical variables, as appropriate. Suicidal ideation was defined as a nonzero response to item 9 of the PHQ-9. Daily nutrient intake values were derived from a 24-h dietary recall and analyzed using the Korean Food Composition Table (9th revision).

**Table 2:** Association between dietary β-carotene intake and suicidal ideation among cancer survivors: results from multivariable logistic regression models.

Exposure	Model 1	*p*	Model 2	*p*	Model 3	*p*
	OR (95% CI)		OR (95% CI)		OR (95% CI)	
β-carotene intake (per 1000 µg increase)	0.61 (0.45, 0.83)	0.002	0.64 (0.47, 0.87)	0.004	0.68 (0.50, 0.93)	0.015
Q1	Reference		Reference		Reference	
Q2	0.67 (0.37, 1.23)	0.197	0.72 (0.40, 1.32)	0.294	0.64 (0.30, 1.37)	0.248
Q3	0.42 (0.21, 0.82)	0.012	0.47 (0.24, 0.94)	0.032	0.53 (0.23, 1.24)	0.145
Q4	0.55 (0.29, 1.02)	0.059	0.60 (0.32, 1.14)	0.117	0.84 (0.38, 1.86)	0.670
*p* for trend	0.057		0.145		0.429	
Age						
<60	0.58 (0.40, 0.84)	0.004	0.58 (0.40, 0.84)	0.004	0.58 (0.38, 0.89)	0.013
≥60	0.75 (0.58, 0.97)	0.026	0.67 (0.39, 1.16)	0.151	0.79 (0.57, 1.11)	0.169
Sex						
Men	0.68 (0.44, 1.06)	0.087	0.68 (0.44, 1.06)	0.087	0.66 (0.42, 1.06)	0.084
Women	0.56 (0.37, 0.85)	0.006	0.61 (0.40, 0.93)	0.022	0.96 (0.67, 1.39)	0.839
Time since cancer diagnosis						
≤5 y	0.98 (0.73, 1.31)	0.878	1.02 (0.76, 1.36)	0.921	1.16 (0.79, 1.70)	0.452
>5 y	0.49 (0.32, 0.75)	0.001	0.51 (0.33, 0.78)	0.002	0.68 (0.50, 0.93)	0.015

Note. Odds ratios (ORs) and 95% confidence intervals (CIs) were estimated using logistic regression models. Model 1: Unadjusted (crude); Model 2: Adjusted for age and sex; Model 3: Fully adjusted for age, sex, marital status, educational level, household income level, smoking status, drinking status, aerobic exercise, resistance exercise, hypertension, dyslipidemia, diabetes mellitus, body mass index (BMI), depression, daily energy intake, vitamin A, vitamin B1, vitamin B2, vitamin B3, vitamin C, vitamin D, vitamin E, folate, and time since cancer diagnosis. β-carotene intake was analyzed both as a continuous variable (per unit increase) and as a categorical variable divided into quartiles (Q1–Q4). The *p* for trend was calculated by treating the median value of each quartile as a continuous variable in the model. Stratified analyses were conducted by age, sex, and time since cancer diagnosis.

## Data Availability

The data analyzed in this study is openly accessible via the KNHANES website (https://knhanes.kdca.go.kr/knhanes/eng/main.do, accessed on 4 June 2024).

## Supplementary Materials

The following supporting information can be downloaded at: https://www.mdpi.com/article/10.3390/nu18101567/s1, Figure S1: Adjusted odds ratios for suicidal ideation among cancer survivors: multivariable logistic regression analysis; Figure S2: Restricted cubic spline analysis of the nonlinear association between β-carotene intake and suicidal ideation, stratified by (A) age, (B) sex, and (C) time since cancer diagnosis; Figure S3. Distribution of β-carotene intake with a rug plot; Table S1: Variance inflation factors (VIFs) for variables included in the multivariable logistic regression model.

## Abbreviations

The following abbreviations are used in this manuscript:

ANOVA	Analysis of variance
BDNF	Brain-derived neurotrophic factor
BMI	Body mass index
CI	Confidence interval
HDL	High-density lipoprotein
IRB	Institutional Review Board
KDCA	Korea Disease Control and Prevention Agency
KFCT	Korean Food Composition Table
KNHANES	Korea National Health and Nutrition Examination Survey
LDL	Low-density lipoprotein
OR	Odds ratio
PHQ-9	Patient Health Questionnaire-9
RCS	Restricted cubic spline
SD	Standard deviation
TNF-α	Tumor necrosis factor-alpha
VIF	Variance inflation factor
WHO	World Health Organization
