# Supplementary material for: U-Shaped Association Between β-Carotene Intake and Suicidal Ideation in Cancer Survivors: Evidence from a Nationally Representative Sample
Source: Nutrients. 2026 May 14;18(10):1567. doi: 10.3390/nu18101567 (PMC13209203; doi:10.3390/nu18101567)

Supplementary Table S1. Variance inflation factors (VIFs) for variables included in the multivariable logistic regression model.

Variable	VIF
Age	2.09
Sex	2.88
Household income	1.37
Educational level	1.62
Marital status	1.10
Smoking status	2.36
Alcohol consumption	1.43
Aerobic exercise	1.15
Resistance exercise	1.34
Total energy intake (kcal)	4.55
Body mass index (BMI)	1.28
Hypertension	1.36
Diabetes (HbA1c-based)	1.14
Hyperlipidemia	1.23
Protein intake (quartile)	4.77
Fat intake (quartile)	3.29
Carbohydrate intake (quartile)	3.89
β-Carotene intake (quartile)	1.46
Retinol intake (quartile)	1.74
Niacin intake (quartile)	3.51
Folate intake (quartile)	2.68
Vitamin C intake (quartile)	1.71
Vitamin D intake (quartile)	1.51
Vitamin E intake (quartile)	2.97
Vitamin B1 intake (quartile)	1.93
Vitamin B2 intake (quartile)	2.01
Time since cancer diagnosis	1.25
Depression (PHQ-9 ≥10)	1.31

Note. VIF: Variance Inflation Factor. All values were below 10, indicating no severe multicollinearity among covariates.

Supplementary Figure S1. Adjusted odds ratios for suicidal ideation among cancer survivors: multivariable logistic regression analysis.

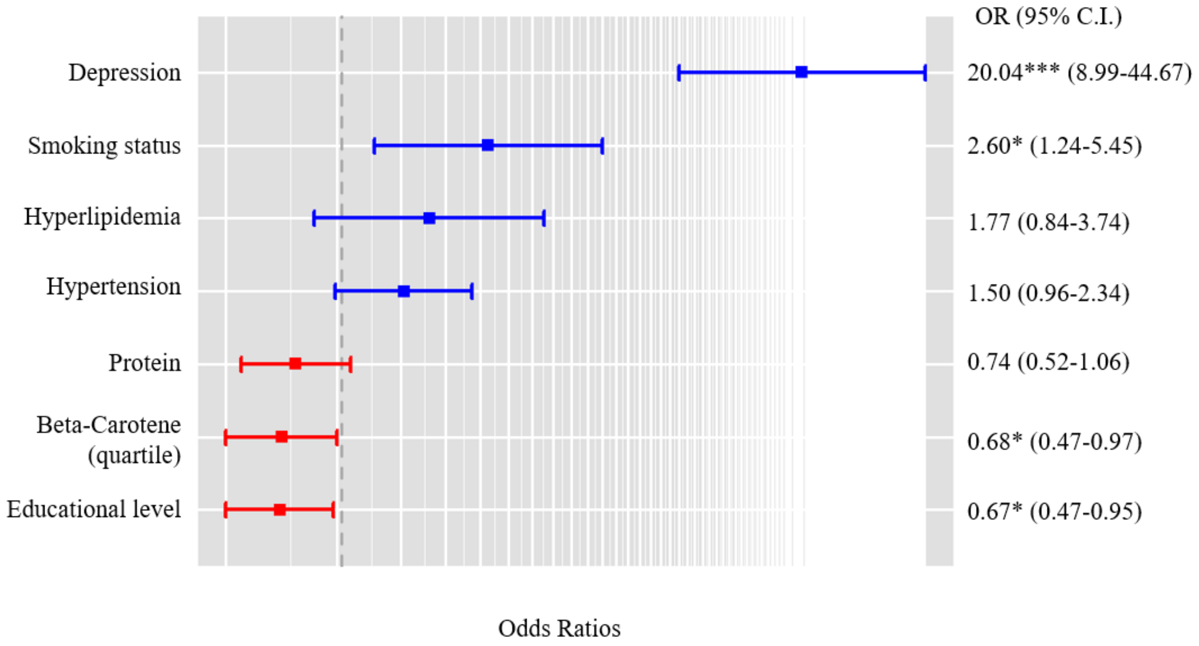

*Note: Estimates adjusted for age, sex, marital status, educational level, household income level, smoking status, drinking status, aerobic exercise, resistance exercise, hypertension, dyslipidemia, diabetes mellitus, depression, body mass index, daily energy intake, vitamin A, vitamin B1, vitamin B2, vitamin B3, vitamin C, vitamin D, vitamin E, folate, and time since cancer diagnosis.

*$p < 0.05$, **$p < 0.01$, ***$p < 0.001$.

Supplementary Figure S2. Restricted cubic spline analysis of the nonlinear association between β-carotene intake and suicidal ideation, stratified by (A) age, (B) sex, and (C) time since cancer diagnosis.

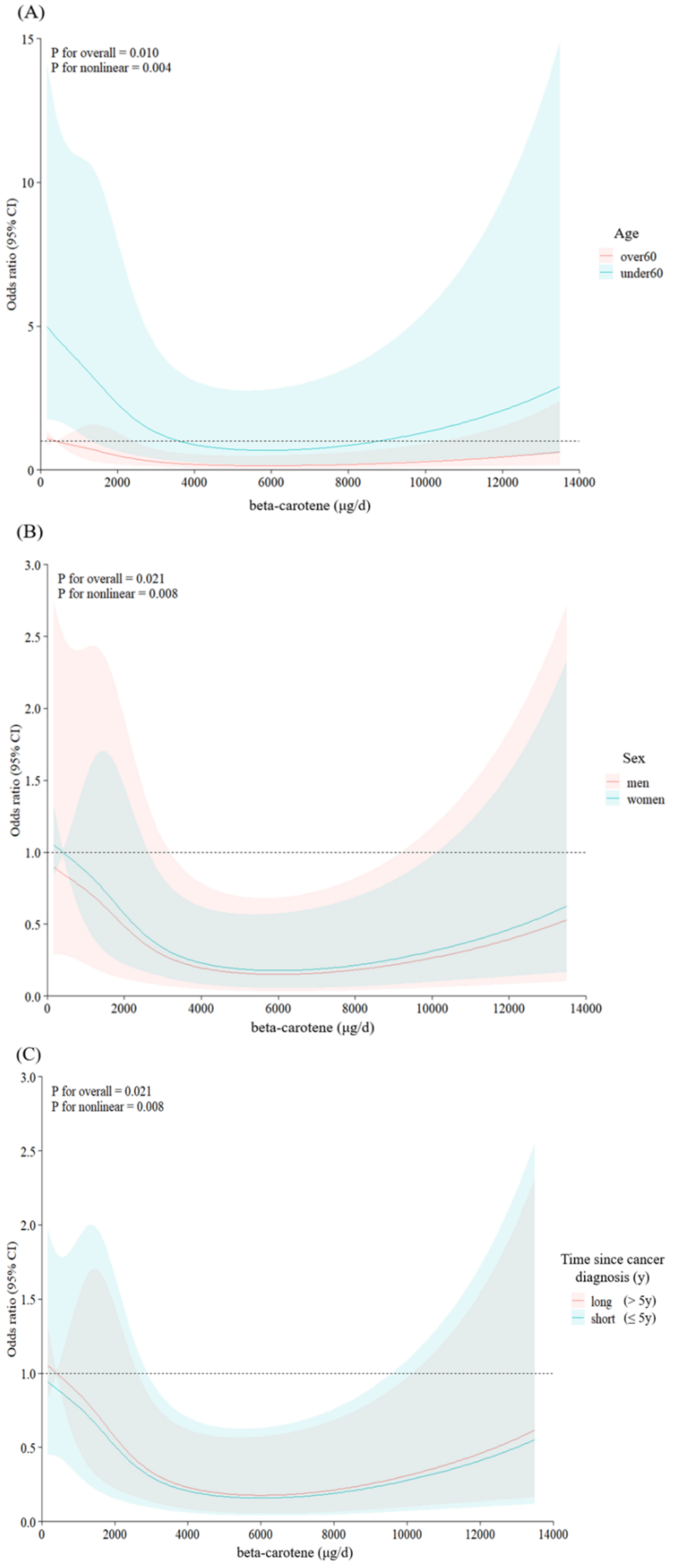

Note. Models were fully adjusted for age, sex, marital status, educational level, household income level, smoking status, drinking status, aerobic exercise, resistance exercise, hypertension, dyslipidemia, diabetes mellitus, depression, body mass index, daily energy intake, vitamin A, vitamin B1, vitamin B2, vitamin B3, vitamin C, vitamin D, vitamin E, folate, and time since cancer diagnosis. Shaded areas represent 95% confidence intervals.

Supplementary Figure S3. Distribution of β-carotene intake with a rug plot

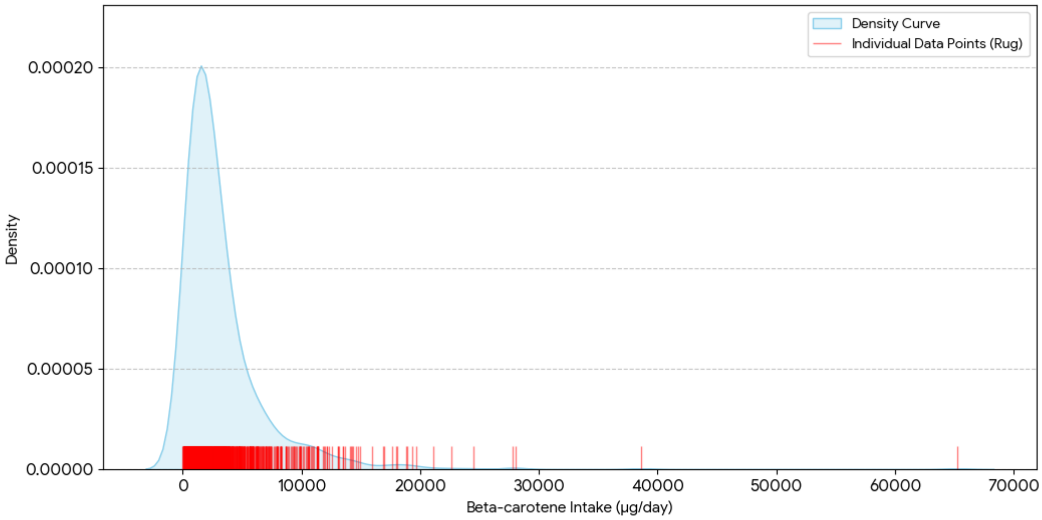

Supplement: Supplementary file 1 [file nutrients-18-01567-s001.zip › nutrients-4246857-supplementary.pdf]
